# Aqueous Self-Sorting in Extended Supramolecular Aggregates

**DOI:** 10.3390/ijms14011541

**Published:** 2013-01-14

**Authors:** Christina Rest, María José Mayoral, Gustavo Fernández

**Affiliations:** Institut für Organische Chemie and Center for Nanosystems Chemistry, Universität Würzburg Am Hubland, 97074 Würzburg, Germany; E-Mails: rest@chemie.uni-wuerzburg.de (C.R.); maria.mayoral-munoz@uni-wuerzburg.de (M.J.M.)

**Keywords:** supramolecular chemistry, self-sorting, self-assembly, co-aggregation

## Abstract

Self-organization and self-sorting processes are responsible for the regulation and control of the vast majority of biological processes that eventually sustain life on our planet. Attempts to unveil the complexity of these systems have been devoted to the investigation of the binding processes between artificial molecules, complexes or aggregates within multicomponent mixtures, which has facilitated the emergence of the field of *self-sorting* in the last decade. Since, artificial systems involving discrete supramolecular structures, extended supramolecular aggregates or gel-phase materials in organic solvents or—to a lesser extent—in water have been investigated. In this review, we have collected diverse strategies employed in recent years to construct extended supramolecular aggregates in water upon self-sorting of small synthetic molecules. We have made particular emphasis on co-assembly processes in binary mixtures leading to supramolecular structures of remarkable complexity and the influence of different external variables such as solvent and concentration to direct recognition or discrimination processes between these species. The comprehension of such recognition phenomena will be crucial for the organization and evolution of complex matter.

## 1. Introduction

Self-organization, self-sorting and selection processes have been essential since the origin of life on Earth and continue to play a key role in most of the fields of our everyday life. Consider, for instance, that human beings form clubs based on common interests, choose a partner out of a wide variety of potential candidates, or turn down offers when we consider them inappropriate.

This extraordinary degree of selectivity takes place likewise at the nano- or microscopic scale in the vast majority of recognition processes between natural or biological macro(molecules) [1]. Perhaps the most extraordinary high-fidelity recognition process in nature is the DNA replication [2]. During this process, three billion nitrogenous bases are duplicated with an absolute minimum of mistakes, which ultimately enables the biological inheritance in living organisms [3]. Other sophisticated structures such as microtubules [4,5], are made up of dimers of two different globular proteins (α- and β-globulin) that self-assemble into longer filaments [6]. This co-assembly process can only take place after the units of α- and β-globulin self-discriminate.

**Figure 1:**
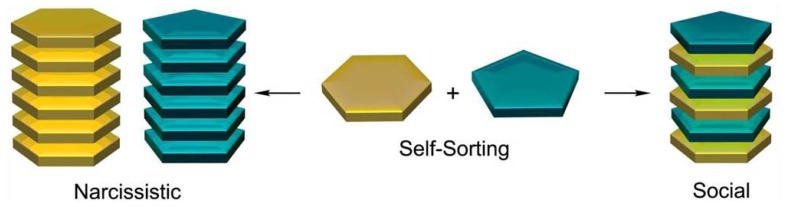
Narcissistic and social self-sorting phenomena in multicomponent mixtures.

In contrast to most natural systems, the behavior of artificial chemical substances, complexes or aggregates in multicomponent mixtures and the creation of co-assembled supramolecular structures have been scarcely investigated [7,8]. However, the extraordinary development of modern analytical techniques in the last decade has brought about the emergence of the field of *self-sorting* [9], which investigates how individual molecules or aggregates respond in the presence of different surrounding species. Molecules or aggregates that show affinity for like species give rise to *narcissistic self-sorting* [10] (also called *self-recognition*), whereas the discrimination of equal molecules and the binding between unlike species can be termed *social self-sorting* [11,12] or *self-discrimination* (Figure 1). The comprehension of this “molecular programming” in aqueous mixtures of artificial aggregates is a prerequisite for a better understanding of the self-assembly pathways in natural systems and might ultimately lead to self-assembled structures of unprecedented size and shape.

**Scheme 1:**
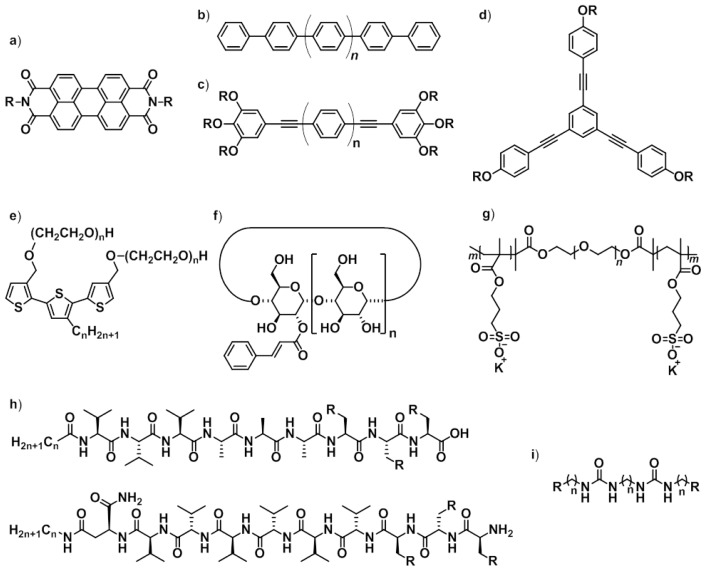
Building blocks that undergo self-sorting processes in aqueous media described in this review.

In this mini-review, we will show the most relevant strategies that have been exploited in recent years to create independent or co-assembled structures upon self-sorting of two or more interacting molecules in aqueous medium. We have classified the examples by taking into account the non-covalent interaction(s) that drive the aggregation phenomena (Scheme 1), whereas the influence of different external variables (solvent, temperature, concentration, pH, *etc*.) on the self-sorting processes will also be discussed.

## 2. Self-Sorting Processes in Extended Supramolecular Aggregates in Water: Influence of Hydrophobic Effect, Intermolecular Interactions and External Variables

Important parameters to consider when analyzing recognition processes in aqueous medium are the exceptional structure and properties of water. Unlike conventional organic solvents, water provides a unique and particular scenario in which most non-covalent forces are strongly influenced. Water molecules are arranged into an infinite dynamic network of hydrogen bonds with a localized structure [13]. This organization is responsible for the deviation of a variety of physical properties but, more importantly, for the *hydrophobic effect*: water molecules are predisposed to form a cage around very non-polar solutes to minimize solvent-solute interactions [14], which is responsible for the tight interaction among non-polar solutes in aqueous media. On the other hand, polar substances are strongly solvated by water and, in most cases, participate in the hydrogen-bonding network. This contraposition in the stabilization of different non-covalent interactions will be a fundamental parameter to control for the successful design of self-assembled structures in aqueous solutions.

In the following sections, we have classified the examples described in the literature depending on the intermolecular force(s) that show a major contribution to the stability of the self-sorted architectures. Although hydrophobic forces play a significant role in most of the examples reported, other secondary interactions such as electrostatic forces and hydrogen bonding can also drive recognition processes if the influence of the hydrophobic effect is reduced.

### 2.1. Self-Sorting Systems Driven by Hydrophobic and π–π Interactions

**Figure 2:**
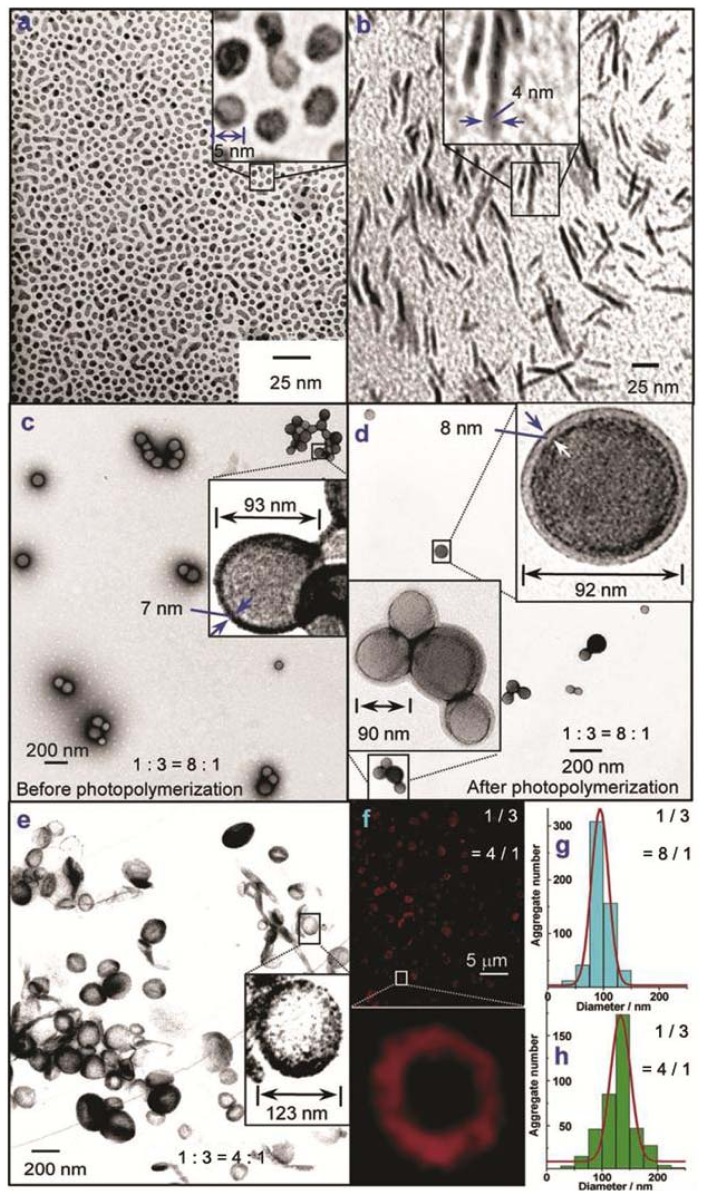
TEM images of self-assembled PBI **1** (**a**), PBI **4** (**b**), (**c**–**d**) co-aggregates of PBI **1** and PBI **3** in a 8:1 molar ratio; TEM (**e**) and confocal fluorescence images (**f**) of co-aggregates of PBI **1** and PBI **3** in a 4:1 molar ratio in THF-containing water (2%, *v*/*v*). Aggregate size distribution of the vesicular co-aggregates ([PBI **1**]/[PBI **3**] = 8/1) (**g**) and ([PBI **1**]/[PBI **3**] = 4/1) (**h**). [PBI **1**] = 0.5 mg mL^−1^ (4.32 × 10^−4^ M); [PBI **4**] = 0.5 mg mL^−1^. Reprinted with permission from [15]. Copyright 2007 American Chemical Society.

**Scheme 2:**
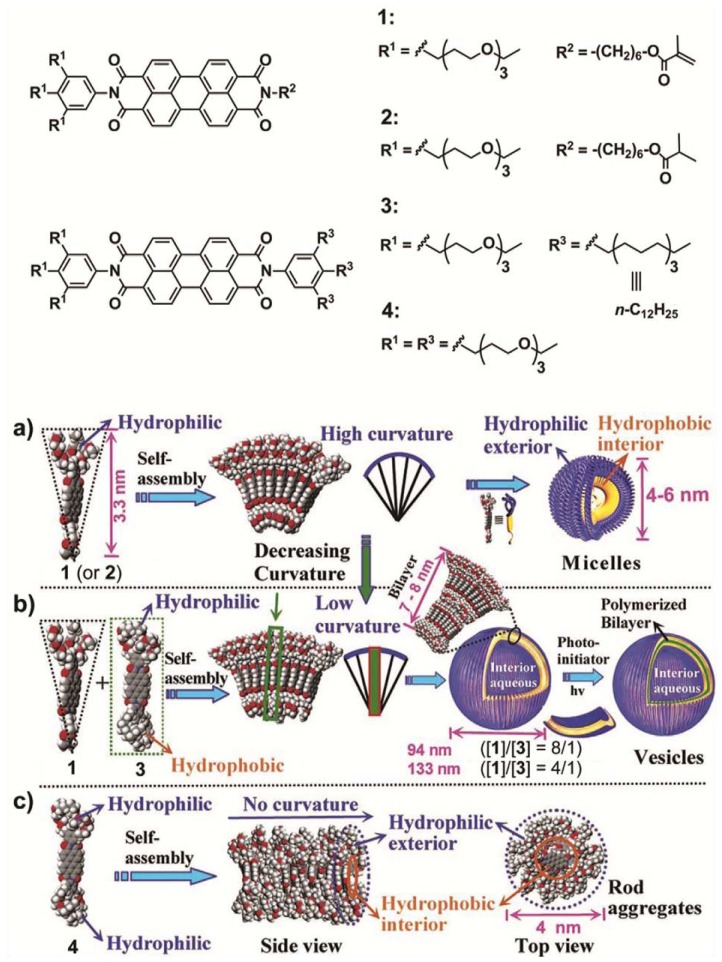
Molecular structures of **1**–**4** and schematic illustration of their self-assembly. (**a**) Micellar aggregates from wedge-shaped perylene bisimides (PBIs) **1**; (**b**) Bilayer vesicles from the co-assembly of PBI **1** and dumbbell-shaped PBI **3**; (**c**) Rod aggregates from dumbbell-shaped PBI **4**. Reprinted with permission from [15]. Copyright 2007 American Chemical Society.

Würthner and co-workers designed four different wedge- and dumbbell-shaped amphiphilic perylene bisimides (PBIs) substituted with a variable number of alkyl and/or polar ethyleneglycol substituents and investigated the self-sorting behavior in a mixture of two of them in aqueous solution [15]. Wedge-shaped PBI **1** and **2** self-assemble into spherical micelles with a diameter of 4–6 nm in THF-containing water (2% *v*/*v*), as visualized by transmission electron microscopy (TEM) studies (Scheme 2a and Figure 2a). This aggregation is driven by strong hydrophobic and aromatic interactions between the hydrophobic PBI cores supported by hydrophilic forces between the glycol chains, giving rise to fluorescent micellar objects with high curvature. In contrast to the behavior of derivatives **1** and **2**, dumbbell-shaped PBI **4** self-assembles into rod-like assemblies driven by π–π and hydrophobic interactions between the PBI cores in a preferred direction with a minimum of curvature (Scheme 2c and Figure 2b). As a result, stiff aggregates with a regular diameter of 4 nm and several tens of nanometers long are formed (see model in Scheme 2c). The authors then questioned what would happen if a wedge-shaped PBI **1** and a dumbbell-shaped PBI **3** were mixed in different ratios (Scheme 2b). Surprisingly, for the mixtures of **1**/**3** in THF-containing water (2% *v*/*v*) with ratios between 8:1 and 4:1 vesicles with average diameters of 94 nm and a bilayer membrane (7–8 nm) could be observed in TEM that exhibited an increasing diameter for higher contents of **3** (Figure 2c,e). The varying size could be explained by taking into account that the higher content of dumbbell-shaped molecules caused a more demanding volume of hydrophobic moieties and consequently the arrangement between the building blocks has a lower curvature. These vesicular compartments have been more recently exploited to encapsulate donor bispyrene moieties in their interior [16]. The donor-loaded vesicles display pH-dependent fluorescence resonance energy transfer from the encapsulated donors to the bilayer dye membrane, providing ultrasensitive pH information on their aqueous environment with fluorescence color changes covering the whole visible light range. These findings provide access to multifunctional nanocontainers with the ability to encapsulate a broad variety of guest molecules.

**Scheme 3:**
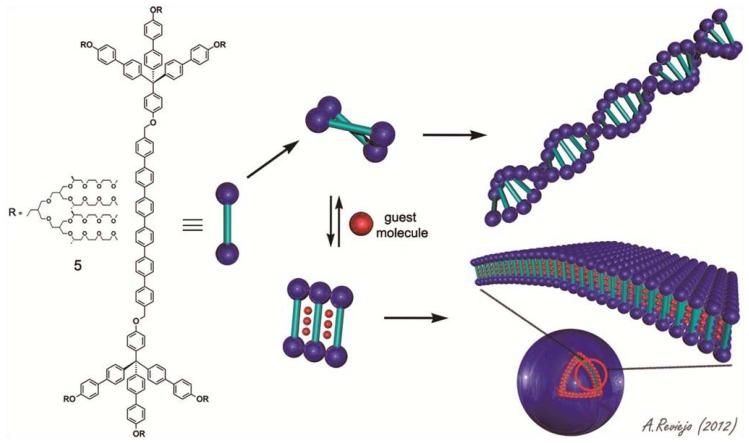
Molecular structure of **5** and representation of the reversible transformation of helical fibers into spherical capsules upon addition of guest molecules. Adapted with permission from [18]. Copyright 2006 Wiley-VCH.

The research group of Myongsoo Lee has devoted intensive studies towards the creation of highly-organized supramolecular architectures in aqueous medium upon self- or co-assembly of a wide variety of amphiphilic systems featuring large hydrophobic segments [17]. In an elegant example, these authors synthesized an amphiphilic dumbbell-shaped molecule (**5**) consisting of a hexa-para-phenylene rod segment and aliphatic polyether dendrons based on a tetrahedral core and investigated whether the addition of aromatic guest molecules would influence the self-assembly behavior (Scheme 3) [18]. The aggregation of amphiphile **5** was first investigated through fluorescence experiments. In a “good” solvent like chloroform, **5** shows two emission maxima at 381 and 400 nm. However, the emission maximum in water is quenched and red-shifted compared to that in chloroform, which is indicative of the aggregation of the hydrophobic rod segments.

**Figure 3:**
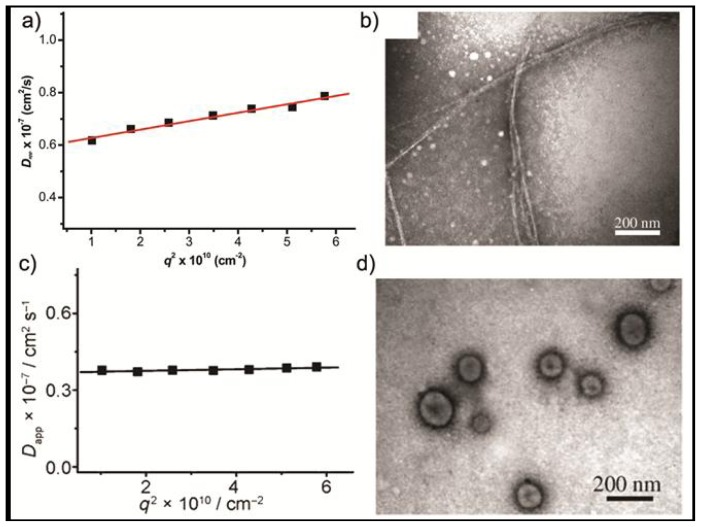
(**a**) Angular dependence (*q* is the scattering vector) of the apparent diffusion coefficient, *D_app_*, for the helical cylinders in aqueous solution (*C* = 0.1 gL^−1^); (**b**) TEM image of **5** with negative staining; (**c**) Angular dependence of *D_app_vs. q^2^* for spherical aggregates; (**d**) TEM image for the spherical capsules with negative staining. Reprinted with permission from [18]. Copyright 2006 Wiley-VCH.

Dynamic light scattering (DLS) experiments on aqueous solutions show an aggregate size of around 60 nm with a slope of the angular dependence of the apparent diffusion coefficient (*D*_app_) of 0.03, suggesting the formation of anisotropic objects (Figure 3a). This was further confirmed by TEM imaging, in which helical cylinders with a uniform diameter of about 10 nm can be observed (Figure 3b). In these aggregates, the elongated aromatic segments stack on top of each other with a slight rotation so that the sterical hindrance between the bulky dendrons is alleviated, while the dendritic substituents are oriented at the periphery exposed to the aqueous environment. By addition of aromatic guest molecules such as 4-bromo-nitrobenzene to an aqueous solution of **5**, a structural rearrangement of the aggregates takes place. In contrast to the behavior of **5** in isolation, the gradient of the slope of the angular dependence of *D*_app_ is approximately zero, which suggests the formation of spherical aggregates (Figure 3c). TEM experiments reveal that the addition of 4-bromo-nitrobenzene induces a parallel orientation of the rod segments in which the guest molecules are intercalated between the hydrophobic units (Scheme 3). In this way a spherical capsule consisting of a unilamellar membrane was formed (Figure 3d). Evidence supporting the formation of hollow capsules was provided by the encapsulation of the hydrophilic fluorescent guest Calcein. When Calcein was encapsulated into these structures its fluorescence was suppressed whereas by heating the solution to 60 °C the fluorescence intensity increased indicating the release of the dye molecules.

**Figure 4:**
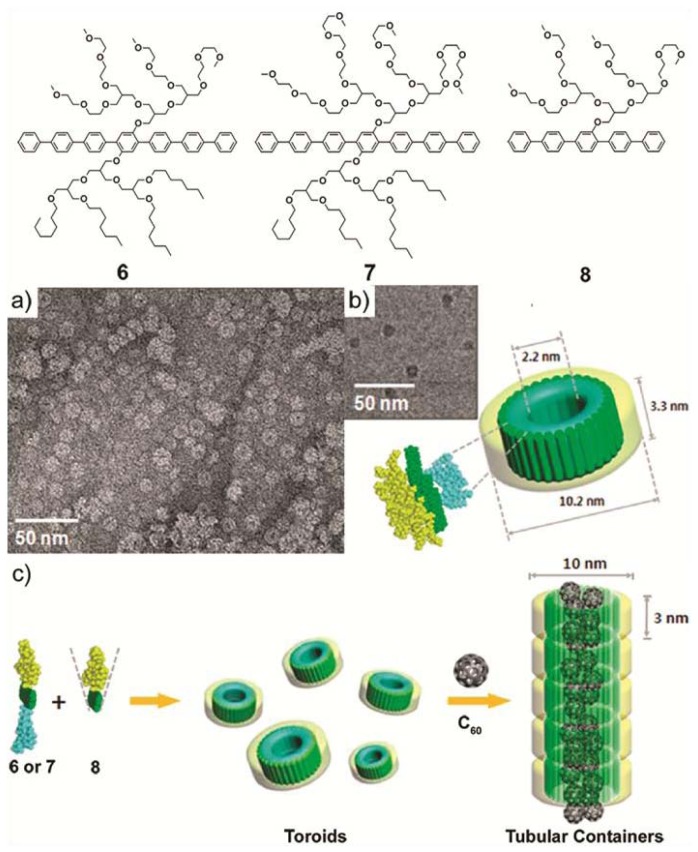
Molecular structures of **6**–**8**. (**a**) TEM image and (**b**) Model and (inset) cryo-TEM image of the ring structure of **6** containing **8** (90 mol% relative to **6**) in aqueous solution; (**c**) Schematic illustration of the stacking of toroids in a 1D manner upon addition of C_60_ guest molecules. Reprinted with permission from [19]. Copyright 2009 American Chemical Society.

In a more recent example, M. Lee and co-workers synthesized two novel laterally substituted amphiphilic molecules (**6** and **7**) that consist of a hepta(*para*-phenylene) core bearing hydrophilic oligoether dendrons on one side and hydrophobic branched heptyl side chains on the opposite side of the aromatic rod [19]. In aqueous solution, **6** and **7** self-assemble forming planar and ribbon-like arrangements, respectively. This structural diversity motivated the authors to investigate whether an increase of the volume fraction of the hydrophilic side chains would induce the formation of more curved structures. To that end, a new amphiphilic derivative **8** featuring a hydrophilic branched chain on only one side was synthesized and its co-assembly with **6** and **7** investigated. DLS experiments indicate that the size of the co-assemblies decreased with increasing content of **8**, whereas TEM pictures demonstrate the reorganization of the molecules from planar sheets for pure **6** to discrete ribbon-like aggregates for the binary mixture (**6:8**) up to a content of **8** of 40 mol%. Further addition of **8** (70 mol%) forced the ribbon structure to eventually convert at 80 mol% into discrete separate toroids (Figure 4a,b). These co-assembled nanostructures consist of a single layer wall where the hydrophobic chains cover the inner part whereas the hydrophilic dendrons are oriented at the periphery. The interior of the co-aggregates of **1** and **8** is hydrophobic and possesses a cavity of 1–2 nm that can be exploited to incorporate [60] fullerene units and solubilize them in aqueous media. First evidence for this encapsulation was provided by fluorescence experiments performed on a mixture of C_60_ and a co-assembled solution of **6** and **8**. The quenching of the fluorescence intensity of the co-assembled mixture upon addition of fullerene demonstrates an effective encapsulation of fullerene units in the cavity of the toroidal objects up to a mole percent of 30%. The encapsulation of fullerenes also influenced the structure of the aggregates. DLS and TEM studies revealed that the aggregates became longer upon addition of C_60_, giving rise to tubular cylindrical structures of 10 nm in diameter and several hundreds of nanometers in length (Figure 4c). The suggested model implies the stacking of the toroids on top of each other stabilized by the fullerene skeleton in the hydrophobic interior. The ability to organize fullerenes at the nanometer scale is an important factor to optimize in the field of optoelectronics [20]. The achievements of Lee and co-workers represent an important contribution with potential application in these fields.

**Figure 5:**
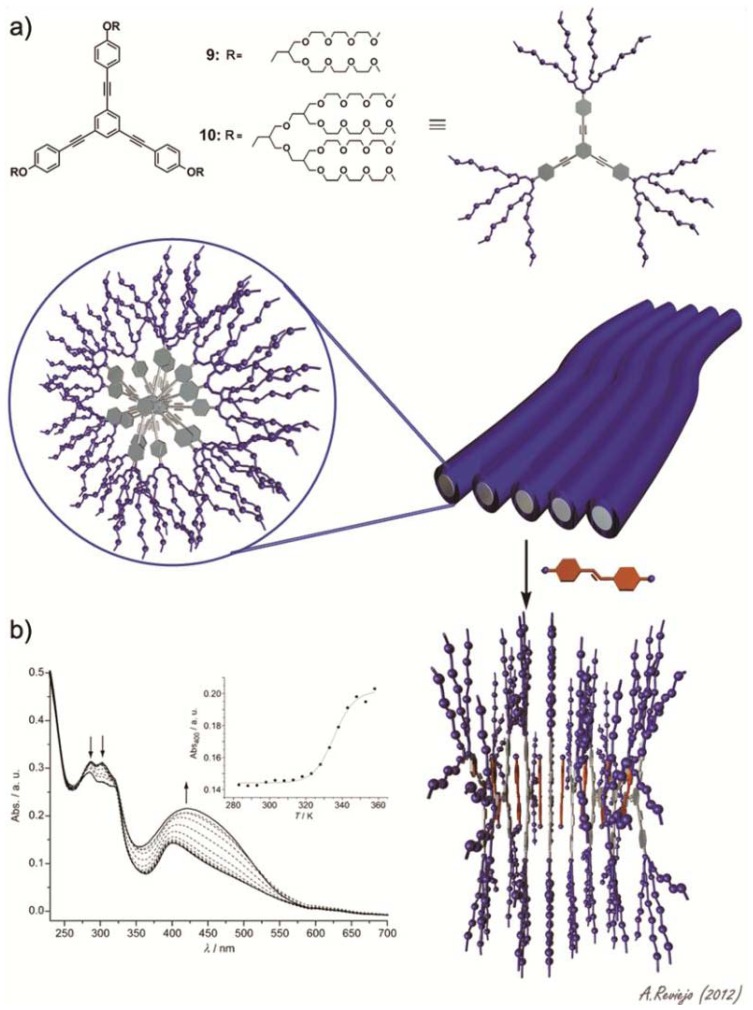
(**a**) Molecular structures of **9** and **10** and schematic representation of their self-assembly into nanofibrillar structures; (**b**) Temperature-dependent UV-Vis spectra of aqueous solutions (~10^−5^ M) of **10** containing ten equivalents of Disperse Orange 3. Arrows indicate the spectroscopic changes upon increasing temperature. The inset depicts the changes in the absorbance at 400 nm as a function of temperature. Reprinted with permission from [21]. Copyright 2010 Wiley-VCH.

Sánchez and co-workers have investigated the self-assembly behavior of two amphiphilic triangular-shaped oligophenyleneethynylene (OPE) derivatives and their co-assembly with the hydrophobic dye Disperse Orange 3 [21]. OPE building blocks are well-known, besides their remarkable optical and electronic properties, for their ability to self-assemble into a wide variety of supramolecular structures [22–31]. The amphiphiles feature a triangular OPE-based aromatic core bearing dendritic glycol chains on each terminal phenyl ring (compounds **9** and **10** in Figure 5a). Concentration- and temperature-dependent experiments in acetonitrile demonstrate the isodesmic aggregation of both amphiphiles with binding constants of ~10^5^ M^−1^. In aqueous solutions, in which the propensity of the amphiphiles to aggregate is higher as a consequence of an increased hydrophobic effect, values of binding constant larger than 10^7^ M^−1^ were predicted. Dynamic (DLS) and static (SLS) light scattering experiments along with TEM imaging demonstrate the formation of nanometer-long fiber-like structures for both dyes. Interestingly, the authors investigated the ability of these fibrillar associates to encapsulate and release a hydrophobic dye, 4-(4-nitrophenyl-azo)aniline (Disperse Orange 3). To that end, an excess of the dye (10 equivalents) was added to an aqueous solution of either **9** or **10** followed by sonication for three hours and removal of the dye excess through filtration. The solutions were then investigated by means of temperature-dependent UV-Vis studies. At room temperature, the absorption corresponding to both the OPE fragments at ~300 nm and the hydrophobic dye (400–550 nm) can be observed, indicating an effective encapsulation of the dye by the fibrillar structures driven by the hydrophobic effect (Figure 5b). Upon increasing temperature, the diagnostic band of the dye gradually increases in intensity, while the maxima at 284 and 303 nm decrease (Figure 5b). The absence of isosbestic points indicates the lack of a defined stoichiometry in the co-assemblies. The spectral changes at 400 nm against temperature are clearly nonlinear (inset in Figure 5b), which is diagnostic of a reversible dye intercalation-release process. This reversible encapsulation-release process anticipates the potential applicability of these systems in drug-delivery processes.

**Scheme 4:**
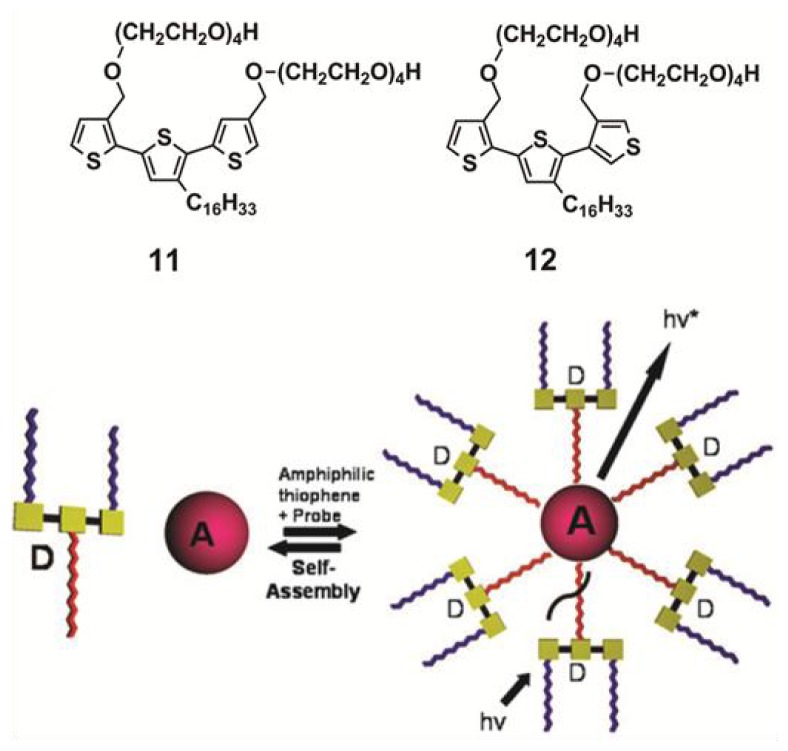
Molecular structures of **11** and **12** and schematic representation of the ET-system that is formed from the donor (D) thiophene amphiphiles (**11** and **12**) and the hydrophobic acceptor (A). Reproduced with permission from [32]. Copyright 2009 The Royal Society of Chemistry.

Van Esch *et al.* have exploited a similar strategy for the encapsulation of suitable hydrophobic acceptor molecules into the interior of micellar associates based on donor amphiphiles, thus creating a stable self-assembled donor-acceptor ET-system [32]. Two amphiphiles (compounds **11** and **12** in Scheme 4) consisting of a hydrophobic terthiophene core substituted with a hydrophobic hexadecane chain in the central ring and hydrophilic tetraethylene glycol chains connected to the peripheral thiophene rings were synthesized. Above 1 mM, amphiphiles **11** and **12** self-assemble in water into spherical and cylindrical micelles, respectively, with diameters of 6 ± 2 nm for **11** and 21 ± 5 nm for **12**, as demonstrated by cryogenic TEM and DLS experiments. More interestingly, the potential of the formed micelles to act as a host for hydrophobic chromophores creating an ET-system was investigated. To that end, two hydrophobic chromophores (Nile Red and Tetraphenylporphyrin) were chosen because of the overlap of their absorption spectra with the emission spectra of the amphiphilic terthiophenes. First evidence suggesting an encapsulation process is provided by the fact that both hydrophobic dyes become soluble in water upon addition of **11** or **12** above their critical micellar concentration. The encapsulation process was followed by fluorescence experiments. A simultaneous quenching of the emission of **11** or **12** upon addition of the corresponding acceptor molecules along with a gradual increase of the emission intensity of the acceptors support the creation of a self-assembling energy transfer system in water between the donor aggregates and the acceptor guest molecules, as depicted in Scheme 4. More recently, Nandi and co-workers have exploited the gelation ability of melamine-quinazoniledione pairs to construct a similar light harvesting hydrogel with FRET emission upon encapsulation of riboflavin units [33].

**Figure 6:**
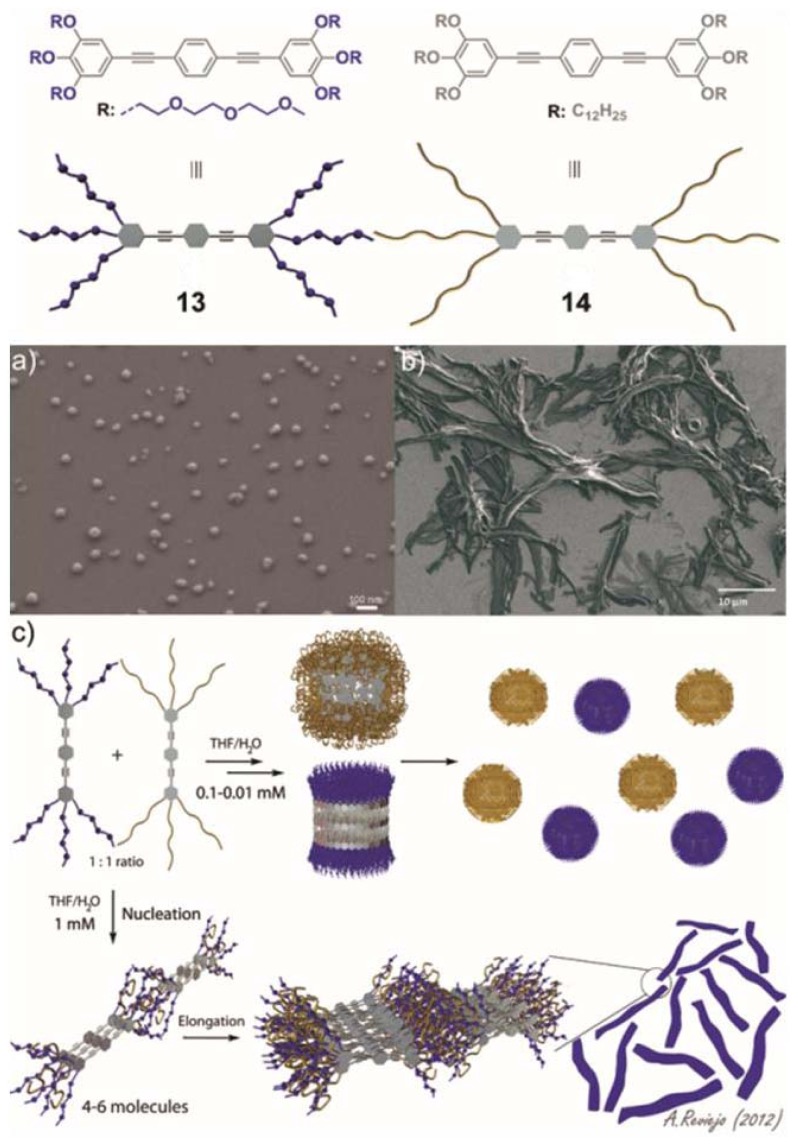
Chemical structures and cartoon representation of OPEs **13** and **14**. SEM images of a 1:1 mixture of **13** and **14** in THF/water at (**a**) 0.1 mM and (**b**) 1 mM on silicon wafer; (**c**) Cartoon representation of the concentration-dependent self-sorting behavior of a 1:1 mixture of **13** and **14** in THF/water (1:1). Narcissistic self-sorting at concentrations between 0.01 and 0.1 mM (top) and social self-sorting at 1 mM (bottom). Reprinted with permission from [34]. Copyright 2012 Wiley-VCH.

In our group, we are particularly interested in the behavior of small synthetic oligophenyleneethynylene (OPE)-based amphiphiles in multicomponent mixtures. Despite that the supramolecular properties of OPE derivatives are relatively well-understood, the ability of directing narcissistic or social self-sorting processes in these systems remains unexplored. Recently, we described the self-assembly of two structurally related OPE derivatives substituted with polar or nonpolar chains (Figure 6) and their narcissistic *vs.* social self-sorting behavior in aqueous media that can be tuned by concentration and solvent changes [34].

The self-assembly mechanisms of OPEs **13** and **14** were studied by temperature-dependent UV-Vis experiments in THF/water mixtures suggesting a non-cooperative (isodesmic) self-assembly process between well-defined species. AFM and SEM imaging confirmed the appearance of spherical micelles with sizes between 3 nm and 10 nm for **13** and diameters ranging from 4 to 50 nm for **14**, which were in agreement with the values observed by DLS. This remarkable propensity of both OPEs to aggregate was exploited to investigate their self-sorting behavior in aqueous mixtures driven by geometrical complementarity and the hydrophobic effect. Self-sorting experiments were carried out in 1:1 THF/water mixtures for an adequate comparison of the behavior of **13** and **14** in isolation and in their mixtures. At relatively low concentrations (0.01–0.1 mM) the 1:1 mixture of both compounds self-assembles into independent spherical micelles (Figure 6a and model shown in Figure 6c top). By simply raising the concentration to 1 mM the equilibrium is shifted towards the formation of social self-sorted systems, giving rise to micrometric-sized ribbon-like aggregates with widths of about 2 μM and lengths of several microns (Figure 6b and model shown in Figure 6c bottom). This unprecedented process is accompanied by a change in the aggregation mechanism from isodesmic to cooperative, as demonstrated by temperature-dependent UV-Vis experiments. The co-aggregates also showed lyotropic liquid crystal behavior—an emergent property of the mixture—that was not observed in any of the compounds in isolation. Our findings demonstrate that subtle changes in certain parameters such as solvent and concentration are efficiently capable of driving the system towards narcissistic or social self-sorting systems.

### 2.2. Self-Sorting Systems Driven by Geometrical Complementarity

**Figure 7:**
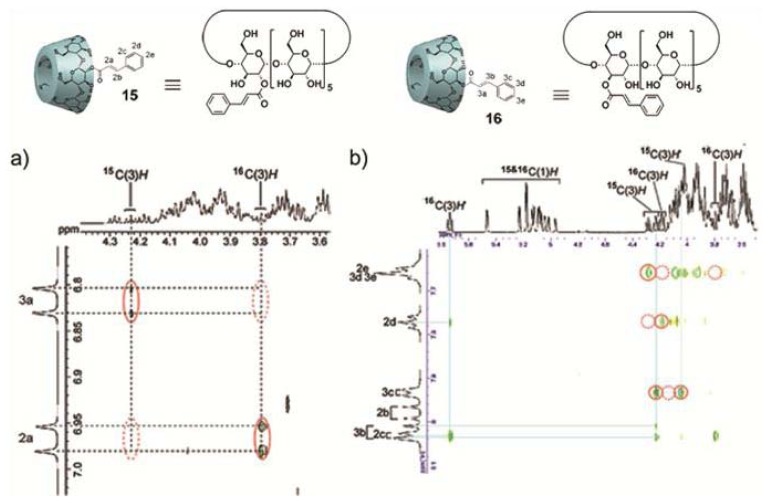

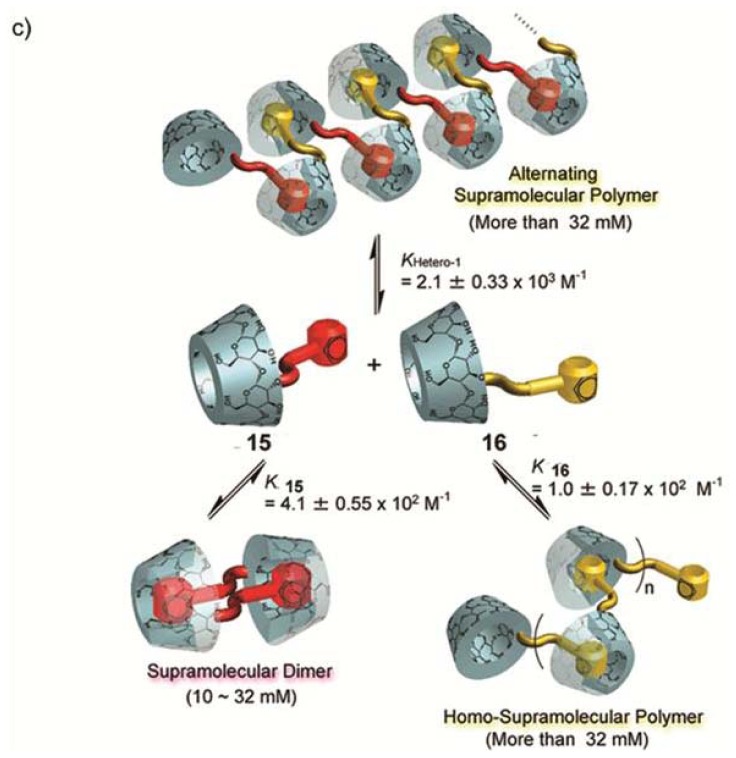
Molecular structures of 2-CiO-α-CD (**15**) and 3-CiO-α-CD (**16**). Two-dimensional ROESY NMR spectra (**a**,**b**) of the mixture of **15** and **16** in D_2_O (32 mM) at 20 °C. The spectrum shows the partial area between inner protons of CiO-α-CDs and olefin protons of the cinnamoyl group. The correlation peaks were observed in the area of solid circles, whereas in the dash circles were not observed. (**c**) Illustration of the formation of supramolecular complexes using CiO-α-CDs. Reprinted with permission from [37]. Copyright 2009 American Chemical Society.

In the previous section, we have shown that strong hydrophobic interactions can efficiently direct either co-assembly processes between amphiphilic molecules with similar geometry or the encapsulation of small aromatic guest molecules in aqueous medium. However, if the weight of hydrophobic interactions is reduced, other intrinsic factors can strongly influence the outcome of a complex mixture. Cyclodextrins (CDs)–a family of compounds made up of sugar units in a cyclic fashion–are archetypal examples in this regard, as they show both a relatively high solubility in water and a great ability to form host-guest complexes with hydrophobic molecules of appropriate size [35,36]. However, in contrast to amphiphiles comprising large aromatic surfaces, their enhanced solubility in water and the presence of well-defined cavities facilitate that small geometrical variations in their molecular structure can lead to dramatic changes in their self-sorting behavior. A clear example illustrating this effect was recently reported by Harada and co-workers [37]. The authors exploited the well-known ability of cyclodextrins to encapsulate cinammoyl groups in their cavity to investigate the recognition and self-sorting phenomena of two isomers of cinammoyl cyclodextrins **15** and **16** that only differ in the orientation of the cinammoyl group relative to the CD unit (Figure 7). Cyclodextrin **15** was found to form a double-threaded dimer (**15**)_2_, as confirmed by 2D ROESY experiments and X-ray crystallographic analysis. These dimers are formed by a mutual fitting of the cinnamoyl unit into the cavity of a CD fragment of a neighboring unit. In contrast, the isomer 3-CiO-α-CD (**16**) self-assembles into extended supramolecular oligomers (**16**)*_n_* above a concentration of 32 mM, as demonstrated by ROESY and pulse field gradient (PFG) NMR experiments. The self-sorting behavior was investigated by mixing both isomers in water. Since the 2-CiO-α-CD (**15**) isomer is moderately soluble in water, the increased solubility in the mixture with 3-CiO-α-CD (**16**) provided first evidence suggesting a co-assembly process. The absence of correlation peaks in the 2D ROESY spectrum between the same isomers in the mixture and the appearance of cross peaks between the olefin and aromatic protons of isomer **15** with the inner protons of isomer **16** demonstrates the formation of an alternating supramolecular polymer and not a self- or random supramolecular complex (Figure 7a–c). These alternating aggregates are formed in a rather cooperative fashion, as suggested by the steep slope of the diffusion coefficients against the concentration of CD units above a certain concentration.

### 2.3. Self-Sorting Systems Driven by Interaction between Charged Groups and/or Hydrogen Bonding Complementarity

**Scheme 5:**
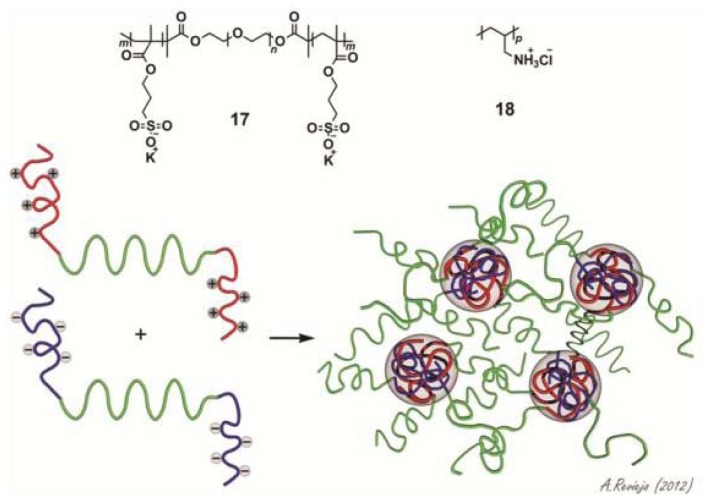
Molecular structures of the negatively charged triblock copolymer PSPMA_28_-PEO_230_-PSPMA_28_ (n~230; m~28) (**17**) and the positively charged homopolymer PAH_160_ (p~160) (**18**) and schematic representation of the formation of reversible gels based on charge-driven assembly. Reprinted with permission from [38]. Copyright 2010 Wiley-VCH.

In contrast to most of the examples reported in the literature that exploit cooperative π–π and hydrophobic interactions to create self-sorted systems, less efforts have been devoted to the design and construction of extended co-aggregated systems based on other secondary interactions. A promising alternative approach in this regard makes use of strong interactions between positively and negatively charged groups. This strategy has the advantage that the introduction of charged groups enhances considerably the solubility in aqueous medium and, if polar substituents are also present, the influence of hydrophobic forces can be neglected. In this regard, Lemmers and co-workers have studied the co-assembly behavior in water of a triblock copolymer bearing a neutral hydrophilic core with two negatively charged terminal groups (**17**) and a homopolymer PAH_160_ bearing positively charged amino functions (**18**) and investigated the influence of different variables (stoichiometry, temperature, ionic strength and pH variations) on the self-sorting processes [38]. Diluted binary mixtures of **17** and **18** at different ratios were studied by DLS. An increase in the scattered light intensity was observed to reach a maximum when the positive and negative charges have been compensated, after which it decreases again. These results suggest the co-assembly of the two compounds forming flowerlike micelles with a hydrodynamic radius of ~20 nm (Scheme 5). At higher concentration, a reversible gelation process takes place in which the micellar structures interconnect into a three-dimensional network. X-ray scattering (SAXS) measurements demonstrate that the micellar cores possess a radius of around 8 nm and an intermicellar distance of around 30 nm (Scheme 5). Different studies revealed that these networks are responsive to changes in concentration, temperature, ionic strength and pH. For instance, rheometry investigations showed that the viscosity of the gel significantly grows with increasing concentration whereas an increase in the temperature showed the opposite effect. In addition, the influence of ionic strength was tested by addition of KCl. With a gradual increase of the salt concentration the scattered light intensity as well as the viscosity of the gel decreased indicating the dissolution of the gel. A similar effect is observed if the pH is raised. Overall, the ability of the hydrogels to respond to different external stimuli makes these materials interesting candidates for different applications in materials science or biomedicine.

**Chart 1:**
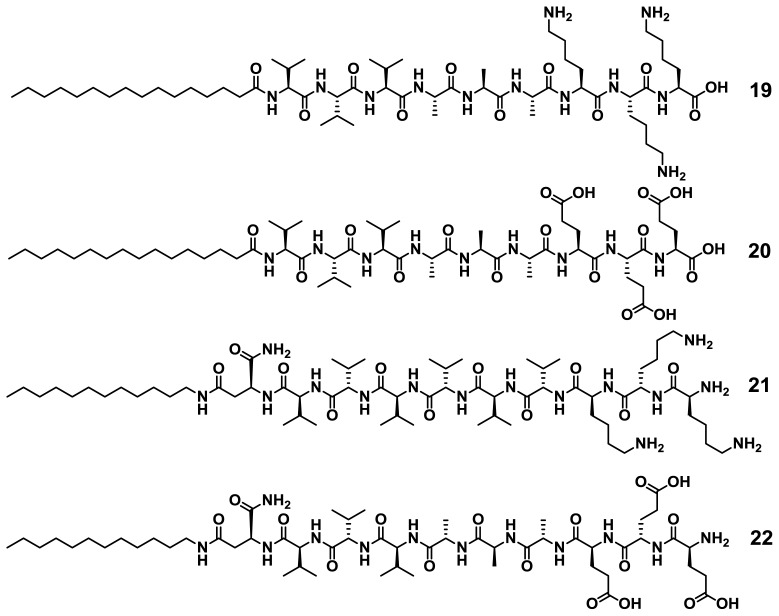
Molecular structures of Peptide Amphiphiles (**19**–**22**). Reprinted with permission from [41]. Copyright 2005 American Chemical Society.

Stupp and co-workers have investigated the self-assembly of a wide variety of peptide amphiphiles (PA) into β-sheet-forming nanofibers driven by hydrogen bonding complementarity and secondary hydrophobic and/or electrostatic interactions [39,40]. Because of their high geometrical similarity and ability to aggregate in aqueous media, such molecules are expected to undergo narcissistic or social self-sorting processes when allowed to interact in multicomponent mixtures. In an elegant example, Stupp and co-workers synthesized four related PAs (**19**–**22**), two positively charged with a triple lysine sequence (**19** and **21**) and two negatively charged with a triple glutamic acid sequence (**20** and **22**) that can lead to co-assembled structures in which the peptide sequence has either normal or reverse polarity (Chart 1) [41].

**Chart 2:**
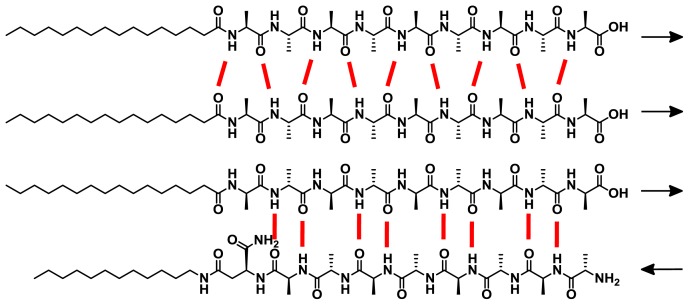
Schematic representation of the aggregation of Peptide Amphiphiles of identical and opposite polarities and their expected arrangements. Reprinted with permission from [41]. Copyright 2005 American Chemical Society.

All peptides form β-sheet structures in isolation upon neutralizing the charges by changing the pH or adding calcium ions. Interestingly, when negatively charged peptides (**20** or **22**) are mixed with complementary charged **19** or **21** residues, mixed nanofibers in which two molecules form a single aggregate structure are created (Chart 2), as shown by circular dichroism (CD) studies. However, when two PAs of similar charge are mixed, less stable β-sheet structures or more disordered conformations are formed due to charge repulsion. Among the supramolecular structures formed upon co-assembly of PAs with identical (**19/20**) or opposite polarities (**20**/**21**), it was shown that the arrangement of hydrogen bonding in the **20**/**21** system is energetically more favorable than that in the **19**/**20** arrangement, which results in an enhanced CD signal. This example clearly demonstrates that the appropriate sequence of hydrogen bonding groups can also efficiently drive co-aggregation processes in water when the molecules possess a good solubility in this medium. More recently, Nilsson and co-workers have investigated the self-sorting behavior in equimolar mixtures of enantiomeric amphipathic peptides [42]. Fluorescence resonance energy transfer (FRET), CD, IR and TEM experiments revealed the formation of β-sheet fibrils formed by an alternating arrangement of l- and d-peptides also driven by hydrogen bonding complementarity in aqueous medium.

**Figure 8:**
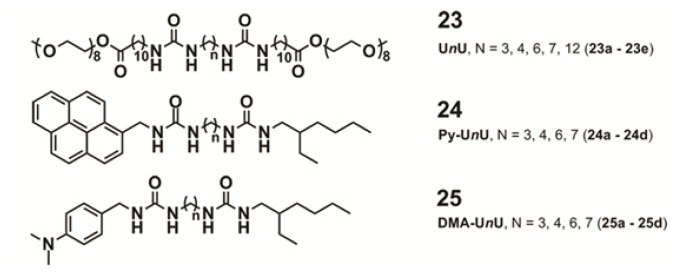

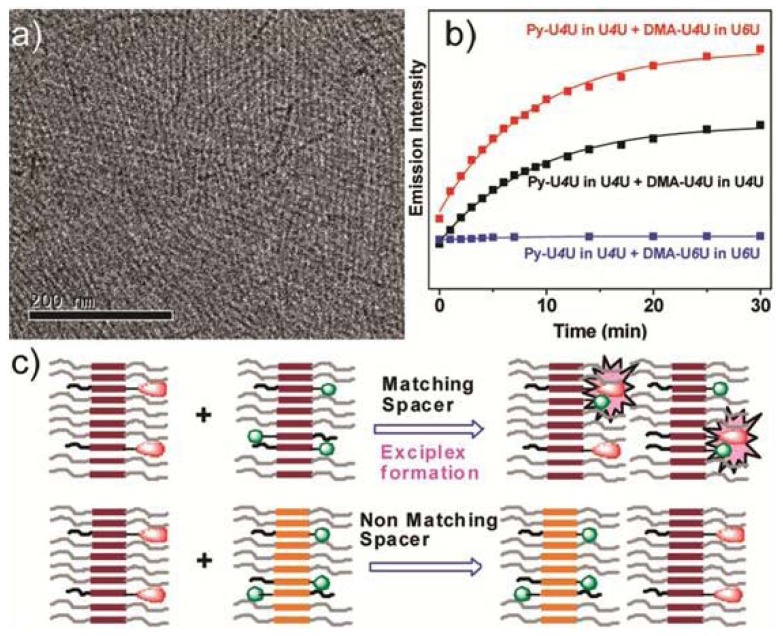
Molecular structures of U*n*U, Py-U*n*U and DMA-U*n*U derivatives (**23**–**25**). (**a**) Cryo TEM images of 1 wt% micellar solutions of U4U (**23b**); (**b**) Intensity of the exciplex band at 520 nm as a function of time upon mixing of micellar solutions containing Py-U4U (**24b**) with micellar solutions containing DMA-U4U (**25b**) or DMA-U6U (**25c**); (**c**) Cartoon representation of the self-sorting phenomena in bisurea rod-like micelles upon addition of matching or nonmatching molecules. Reprinted with permission from [44]. Copyright 2010 American Chemical Society.

Perhaps one of the most illustrative examples highlighting the role of hydrogen bonding complementarity has recently been reported by Sijbesma and co-workers [43,44]. For their investigations, the authors synthesized a series of bisurea-based bolaamphiphiles (U*n*U in Figure 8) composed of two urea groups spaced by alkyl chains of variable length and similar derivatives substituted with fluorescent pyrene and dimethylamino groups (Py-U*n*U and DMA-U*n*U, respectively). These dyes were used as fluorescent probes to investigate whether a co-assembly process takes place, which would result in an exciplex band in the fluorescence spectra if both dyes are in spatial proximity. Bisurea bolaamphiphiles U3U (**23a**), U4U (**23b**), U6U (**23c**) and U7U (**23d**) form micrometer-long cylindrical micelles in water driven by hydrogen bonding between the urea groups, as revealed by Cryo-TEM images (Figure 8a). The self-sorting behavior of U4U (**23b**) was studied by fluorescence spectroscopy using the pyrene probe Py-U4U (**24b**) and the dimethylaniline probe DMA-U4U (**25b**). Upon mixing U4U (**23b**) with either Py-U4U (**24b**) (up to 0.01 eq) or DMA-U4U (**25b**) (up to 0.15 eq), a co-assembly process driven by hydrogen-bonding complementarity takes place in which the pyrene or DMA units are randomly dispersed in the micelles of U4U (**23b**) and no exciplex band is expected. However, upon mixing both solutions (U4U (**23b**)-Py-U4U (**24b**) and U4U (**23b**)-DMA-U4U (**25b**)), a band at 520 nm appeared in the fluorescence spectrum, indicating the molecular contact between Py- and DMA-USU upon co-assembly of three compounds into micellar structures (Figure 8b). Interestingly, when nonmatching micelles U4U (**23b**) (containing 0.01 eq Py-U4U (**24b**)) and U6U (**23c**) (containing 0.15 eq DMA-U6U (**25c**)) were mixed, no exciplex band was observed, indicating that the probes form separate “narcissistic” micelles due to the fact that the distance between the urea groups in U4U (**23b**)-Py-U4U (**24b**) is smaller than that in U6U (**23c**)-DMA-U6U (**25c**) and the hydrogen bonding pattern in the co-assembly cannot take place (Figure 8c). In an even more complex experiment, the authors created up to three separate micellar aggregates by mixing bisurea derivatives with different alkyl spacers. These interesting findings hold potential for the construction of a large number of assemblies that coexist independent from one another in the same medium, providing access to exciting nanomaterials that can mimic the operation of natural systems.

### 2.4. Enzyme-Responsive Self-Sorted Systems

**Figure 9:**
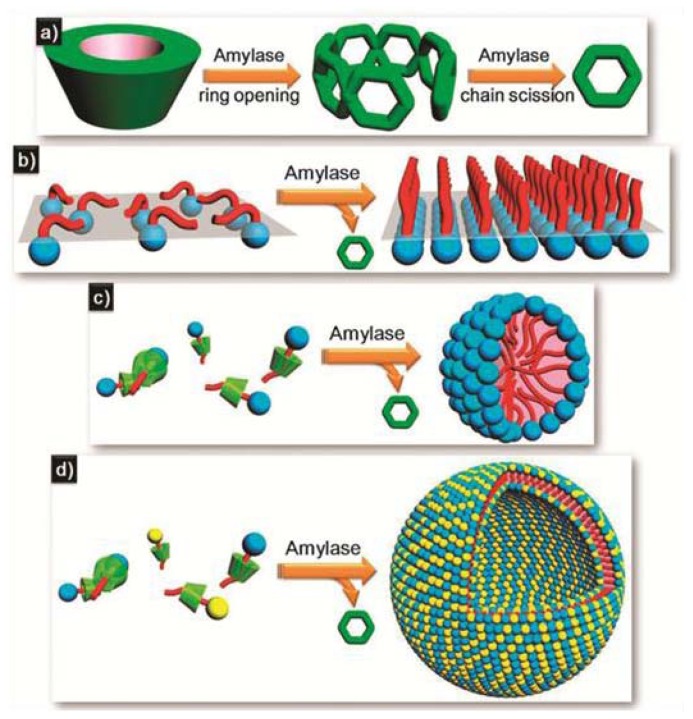
Schematic illustrations of the degradation of β-CD by α-amylase (**a**); enzyme-triggered monolayer formation (**b**); enzyme-triggered micellization (**c**); and enzyme-triggered vesicle formation (**d**). Reproduced with permission from [46]. Copyright 2012 The Royal Society of Chemistry.

A new approach that has gained considerable attention in recent years is the proposal to interface supramolecular objects with biological systems such as enzymes. Enzyme-responsive supramolecular assemblies are a class of smart nanomaterials that undergo macroscopic transitions when triggered by selective catalytic actions of enzymes [45]. Examples include bulk phase transitions (sol-to-gel and gel-to-sol) or the transformation, generation or disassembly of supramolecular structures induced by enzyme addition. Recently, Huang and co-workers have developed a host-guest strategy to construct enzyme-triggered self-sorted systems on the basis of surfactant-cyclodextrin (CD) complexes and α-amylase [46]. CDs are well-known for their ability to encapsulate the hydrophobic moieties of surfactants into their cavities driven by hydrophobic interactions. On the other hand, the enzyme α-amylase has the ability to cleave the α-1,4 linkages between the glucose units of starch molecules including CDs, which ultimately leads to the degradation of the CD units, and the concomitant release and subsequent self-assembly of the surfactant molecules (Figure 9a). The influence of β-CD on the organization of different non-ionic, ionic or zwitterionic surfactants was investigated. Prior to the addition of β-CD, the surfactants are forming adsorption monolayers. However, an excess of β-CD induces the breakdown of the monolayers, which can be again recovered upon subsequent treatment with α-amylase. This enzyme degrades the CD units and triggers the release and subsequent organization of the surfactant molecules into monolayers (Figure 9b). A similar effect is observed for the surfactant tetradecyl dimethylammonium propane sulfonate (TDPS). This molecule self-assembles into micelles in aqueous medium, as revealed by DLS studies. After the addition of an excess of β-CD, the solution scattering is notably reduced, which implies the disassembly of the micelles upon complexation with the CD units. Different dosages of amylase were applied to the β-CD/TDPS co-assembly, the scattering of which is observed to become constant after 24 h. The size distributions calculated by DLS for different dosages are nearly the same and demonstrate the formation of spherical micelles with an average hydrodynamic radius of 2 nm (Figure 9c). Finally, the authors investigated the influence of CD and amylase on vesicular structures formed upon co-assembly of two surfactants, sodium dodecyl sulfate (SDS) and DEAB (dodecyl triethyl ammonium bromide). The SDS/DEAB solution (0.4:0.6 mM) is dominated by vesicular assemblies, which can be degraded upon addition of 8 mM of β-CD. The significant enhancement observed in the scattering of the mixture SDS/DEAB/β-CD upon addition of amylase indicates the formation of large aggregates of several hundreds of nanometers. Cryo-TEM studies reveal the formation of hollow spherical aggregates ranging from 100 to 300 nm, as depicted in Figure 9d. The enzyme α-amylase can therefore trigger the self-assembly of CD-surfactant self-sorted systems into different organized arrangements by degradation of the CD molecules and subsequent release and rearrangement of the surfactant molecules.

**Figure 10:**
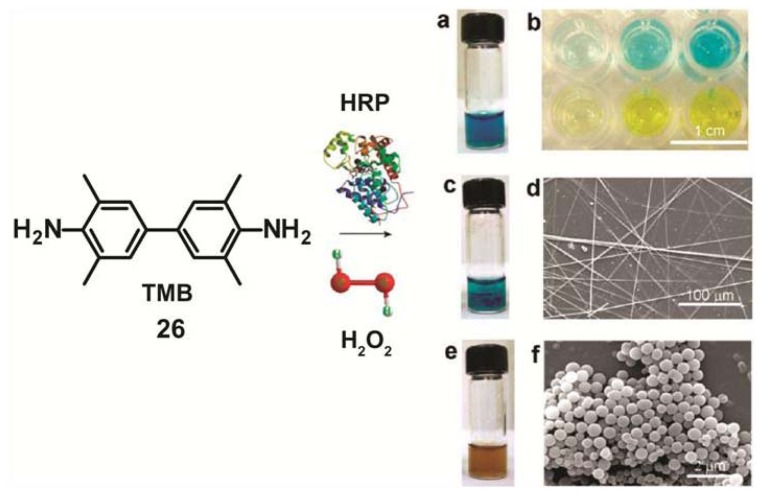
Color reaction and formation of nanostructures in the HRP/H_2_O_2_/TMB system. (**a**) Oxidation of TMB by HRP/H_2_O_2_ in acetate buffer (pH 4.0) produces a blue color; (**b**) Blue color (top row) changes to yellow (bottom row) upon addition of sulfuric acid for typical ELISA analysis; (**c**) Blue nanobelts are formed after allowing the blue solution to react for 24 h at room temperature; (**d**) SEM image of nanobelts; (**e**) Upon mixing additional HRP to the blue mixture of aqueous solution and nanobelts, the color of the system turned brown, accompanied by precipitation of uniform nanoparticles; (**f**) SEM image of nanoparticles. Reprinted or adapted with permission from [47]. Copyright 2011 American Chemical Society.

A similar strategy has been recently developed by Gao and co-workers for the self-assembly and transformation of nanostructures induced by enzymes in a tetramethylbenzidine (TMB)/horseradish peroxidase (HRP)/H_2_O_2_ system [47]. TMB is the most common chromogenic substrate for HRP used as color reagent in enzyme-linked immunosorbent assay (ELISA).The oxidation of TMB (**26**) by HRP/H_2_O_2_ at pH value of 4 produces a blue color that can change to yellow when the reaction is stopped by addition of sulfuric acid (Figure 10a,b). However, when the blue solution reacts for 24 h at room temperature without stopping it with sulfuric acid, nanobelt-like structures with lengths up to 1 mm are formed (Figure 10c,d). More interestingly, if an additional amount of the enzyme HRP is added to this mixture, the color of the system turns brown and ultimately the precipitation of uniform spherical nanoparticles with diameters of ~120–800 nm takes place (Figure 10e,f). These findings indicate that this reaction enables the generation and transformation of different co-assembled structures under control of enzymatic kinetics. The concentration of the enzyme HRP was observed to play a more significant role than the concentration of TMB and H_2_O_2_ in the self-sorting phenomena in this system. These enzyme-responsive materials may find application in smart circuitry and bioresponsive devices.

## 4. Conclusions

In this review, we have shown various strategies utilized by different research groups to construct extended supramolecular aggregates in aqueous medium upon self-sorting of small artificial molecules. Because of the predisposition of water molecules to form a cage around very non-polar solutes, hydrophobic interactions between amphiphilic molecules featuring large aromatic surfaces are expected to be particularly strong in aqueous medium. This has been by far the most common strategy exploited to design aggregates with remarkable size and shape upon co-assembly of two or more aromatic molecules. Although the hydrophobic effect is the major driving force for aggregation of amphiphilic systems in aqueous medium, other non-covalent forces can come into play and drive self-sorting processes if the hydrophobic contribution is reduced, which can be accomplished by enhancing the solubility in water of the interacting molecules. For instance, electrostatic interactions between positively and negatively charged groups are efficiently capable of directing co-aggregate formation in mixtures of molecules featuring a polar, highly water-soluble core. Similar approaches driven by multiple hydrogen bonding can lead to co-aggregate formation in compounds with a high hydrophilia/hydrophobia ratio.

It is not only the hydrophobic effect or the information encoded in the molecular structure of the interacting species that can drive aqueous self-sorting processes, but also external variables (solvent, concentration, temperature, pH, *etc*.) can shift the equilibrium towards the formation of independent or mixed aggregates. In a recent example, an increase in concentration was demonstrated to induce the transformation of two coexisting micellar associates made up of two different molecules into ribbon-like aggregates formed upon co-assembly of both molecules [34]. Although this behavior could be considered routine for self-assembled systems in isolation, the ability to influence particular binding events in multicomponent mixtures is far from being a trivial subject. We hope that a better understanding of the self-sorting phenomena between multiple chemical species coexisting in the same aqueous medium will help us create artificial systems with a degree of complexity closer to that present in nature.
